# A rapid and highly sensitive biomarker detection platform based on a temperature-responsive liposome-linked immunosorbent assay

**DOI:** 10.1038/s41598-020-75011-x

**Published:** 2020-10-22

**Authors:** Runkai Hu, Keitaro Sou, Shinji Takeoka

**Affiliations:** 1 Department of Life Science and Medical Bioscience, Graduate School of Advanced Science and Engineering, Waseda University, Tokyo, Japan; 2 Waseda Research Institute for Science and Engineering, Waseda University, Tokyo, Japan; 3 Institute for Advanced Research of Biosystem Dynamics, Waseda Research Institute for Science and Engineering, Waseda University, Tokyo, Japan

**Keywords:** Nanobiotechnology, ELISA, Fluorescent probes

## Abstract

The enzyme-linked immunosorbent assay (ELISA) is widely used in various fields to detect specific biomarkers. However, ELISA tests have limited detection sensitivity (≥ 1 pM), which is insufficiently sensitive for the detection of small amounts of biomarkers in the early stages of disease or infection. Herein, a method for the rapid and highly sensitive detection of specific antigens, using temperature-responsive liposomes (TLip) containing a squaraine dye that exhibits fluorescence at the phase transition temperature of the liposomes, was developed. A proof-of-concept study using biotinylated TLip and a streptavidin-immobilized microwell plate showed that the TLip bound to the plate via specific molecular recognition could be distinguished from unbound TLip within 1 min because of the difference in the heating time required for the fluorescence emission of TLip. This system could be used to detect prostate specific antigen (PSA) based on a sandwich immunosorbent assay using detection and capture antibodies, in which the limit of detection was as low as 27.6 ag/mL in a 100-μL PSA solution, 0.97 aM in terms of molar concentration. The present temperature-responsive liposome-linked immunosorbent assay provides an advanced platform for the rapid and highly sensitive detection of biomarkers for use in diagnosis and biological inspections.

## Introduction

Enzyme-linked immunosorbent assay (ELISA) tests are common and widely used to detect various biomarkers, including hormones, viral and bacterial antigens, and antibodies produced in response to allergies and infections^1–6^. Conventional ELISA tests have a detection limit of 1 pM^7^; however, in the early stages of many diseases, the concentrations of biomarkers in the serum are estimated to range from 100 aM to1 pM^7–10^. Early diagnosis is critical for treatment regimens to be instigated^7^ and a highly sensitive detection system is needed to enable the measurement of low concentrations.

Recently, a digital counting ELISA method, which uses a micro-chamber loaded with micro-beads that capture target molecules on the surface, and counts the numbers of fluorescent wells by enzymatic amplification associated with image analysis, has attracted much attention^11–14^. These digital counting ELISA methods are based on a mechanism similar to that used in the traditional sandwich ELISA method but have increased sensitivity up to 10,000-fold or higher. Some of these methods can detect biomarkers, such as prostate specific antigen (PSA), at sub-femtomolar concentrations^13,14^. For instance, Rissin et al*.* have designed a digital counting device that uses femtoliter-sized reaction chambers. Each micro-chamber can trap a single bead that can be conjugated with 80,000 antibodies to capture potential target biomolecules. The device loads a total of 400,000 beads and the limit of detection (LOD) of PSA was as low as 250 aM in 25% serum^13^. Kim et al., have designed a similar femto-sized droplet array that has a larger number of microwells, containing more than 1 million beads in total, and the LOD of PSA was increased up to 2 aM^14^. Although those advanced ELISA methods have achieved remarkable improvements toward the detection of low concentrations of samples, some issues still have to be addressed. For example, digital ELISA methods rely on image analysis to distinguish whether the signal is “on” or “off” for each micro-chamber. Therefore, in terms of each microwell, the signal readout is analog in that fluorescence intensity increases as the concentration of the target molecules increases. Consequently, a threshold level is required to be set up to identify positive and negative results, which is subjective and may lead to false-negative results. In addition, to increase the signal-to-noise ratio, the incubation time for the enzymatic digestion of a substrate usually lasts for 5–6 h, which provides no advantage over conventional ELISA tests^13,14^.

Herein, we propose a concept using stimuli-responsive nanoparticles as an ultra-sensitive and fast detection probe, which retains the advantages, but overcomes the drawbacks, of ELISA-based systems. Liposomes are spherical vesicles consisting of one or more types of lipids, in the form of a bilayer. Liposomes can be used as vehicles to carry fluorophores because the physical state of liposomes can be altered by temperature changes. The liposomes change from an ordered gel state to a disordered liquid crystalline state, where the lipid tails become less packed, and more lipids are present in gauche conformations rather than all trans, as the liposomes reach the phase transition temperature^16,17^. The optical properties of the amphiphilic fluorescent dye, SQR22, have a specific response to the liposomal phase transition when the dye is mixed with lipids to form liposomes^18^. In the lipid bilayer membrane in the gel phase, the SQR22 molecules are concentrated to form self-aggregates and the luminescence is quenched because of the aggregation-caused quenching (ACQ) effect^19^. When the liposomes are in a liquid crystalline state, the SQR22 molecules diffuse into the membrane bilayer and emit far-red fluorescence that can be detected. As a result, the liposomes containing SQR22 (SQR liposomes) have temperature-responsive fluorescence characteristics, and the fluorescence intensity of the liposomes markedly changes when the liposomes experience phase transition^18^. A single liposome can incorporate thousands of fluorescent molecules in the hydrophobic region of the bilayer membrane. This large amount of fluorophores in one liposome serves as a signal amplifier to increase the signal-to-noise ratio as the temperature exceeds the gel-to-liquid crystalline phase transition temperature (T_c_) of the liposomes.

In this paper, we report a biomarker detection system using temperature-responsive liposomes containing SQR22 as a fluorescent detection probe for immunosorbent assay (temperature-responsive liposome-linked immunosorbent assay; TLip-LISA). We used PSA as target biomolecule to test the LOD of TLip-LISA. The results suggested that the TLip-LISA has the potential for use in the development of a rapid and highly sensitive detection system for biomarkers.

## Results

### Design of the assay systems

**Figure 1:**
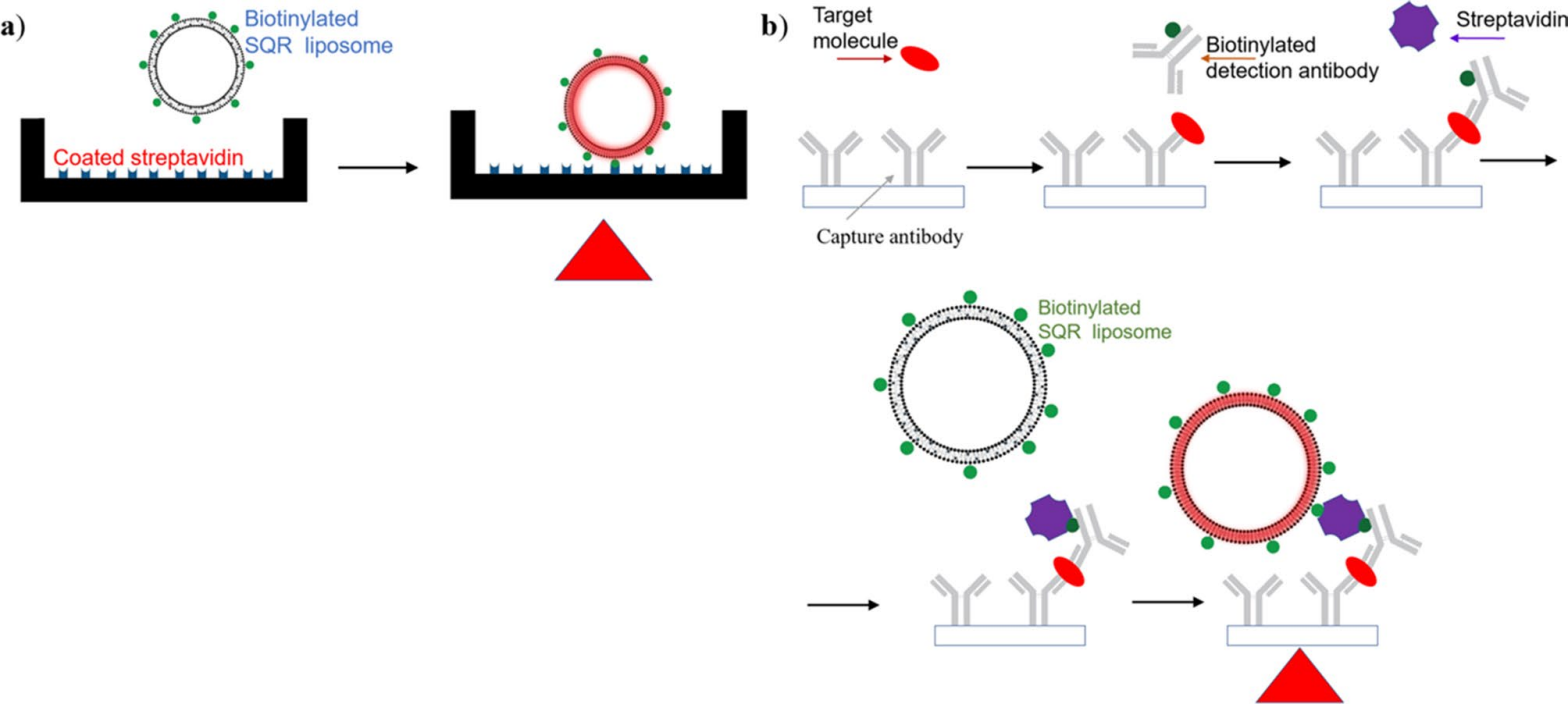
Conceptual drawing of the temperature-responsive liposome-linked immunosorbent assay (TLip-LISA). **(a)** Proof-of-concept study of TLip-LISA using streptavidin as the receptor and biotin as the ligand. **(b)** TLip-LISA for PSA detection.

The concept of the TLip-LISA is illustrated in Fig. 1. We designed a two-step approach for proof of concept and verifying the sensitivity of the TLip-LISA. In the proof-of-concept study, biotin and streptavidin acted as ligand and receptor, respectively. A streptavidin-immobilized plate was used to capture biotin-TLip (TLip incorporated with biotinylated-PEG_2000_-DSPE). The other approach was similar to a sandwich ELISA system, which used TLip with antibodies for detection to replace the enzyme-linked detection antibody. PSA was used as a target molecule, and an anti-PSA antibody-immobilized plate was used to supply antibodies for capture. In this approach, instead of modifying the surface of the liposomes by conjugation with an antibody, we used streptavidin as an intermediate to link a biotinylated antibody for detection with biotin-TLip. A hot plate was used to provide heat for liposomes to reach a liquid crystalline state in both the systems. Instead of reading the absorbance of the dye from the incubated wells after a certain period of time of an enzymatic reaction, we monitored the profiles of the fluorescence intensity from the wells on heating and determined the presence of PSA by the inflection time at which the fluorescence intensity increase rate reaches maximum.

### Characterization of the SQR liposomes

To conduct the assay system shown in Fig. 1, biotin-TLip and PEG-TLip (TLip incorporated with PEG_5000_-DSPE instead of biotinylated-PEG_2000_-DSPE) were prepared using an extrusion method. Both the biotin-TLip and PEG-TLip showed a narrow size distribution where the polydispersity index (PdI) was around 0.1, and the mean diameters of the two liposomes were approximately 90 nm as summarized in Table 1. The negative zeta potential of the liposomes was because of the carboxyl group of 1,5-dihexadecyl-*N*-succiny-L-glutamate (DHSG), which contributed to the enhancement of the liposome stability^21^.The final concentrations of lipids and SQR22 were 2.79 mg/mL and 47.9 µg/mL, and 2.06 mg/mL and 39.2 µg/mL, respectively, for the biotin-TLip and PEG-TLip, respectively. The yields of the lipids and SQR22 of the biotin-TLip after extrusion were determined to be 71.9% and 52.9%, respectively, and 70.2% and 40.2% for the PEG-TLip.

**Table 1:** Characterizations of liposomes.

Samples	Size distribution (nm)	PdI	Zeta potential (mV)
Biotin-TLip	94.5 ± 30.1	0.095	− 13.4
PEG-TLip	90.4 ± 39.6	0.138	− 8.3

**Figure 2:**
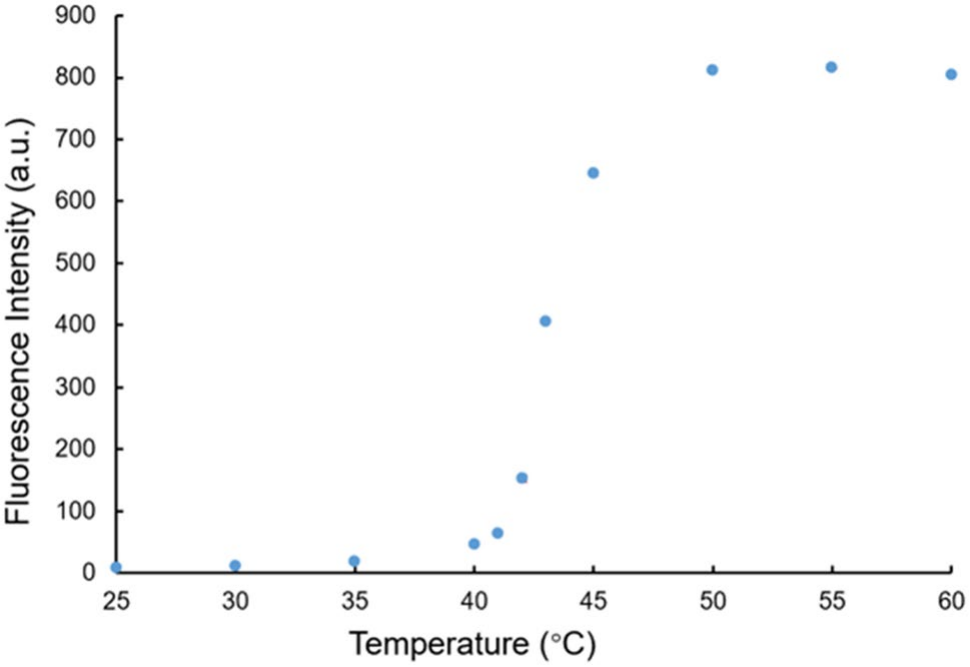
Fluorescence intensity of the biotin-TLip at different temperatures. Data are mean ± standard deviation (SD) acquired from three independent experiments.

As shown in Fig. 2, the fluorescence intensity of the biotin-TLip (the liposome sample was diluted to have a SQR22 concentration of 1 μM, the SQR22/lipid molar ratio was 2.8%) was very weak at room temperature, which was considerably different from the phase transition temperature of 1,2-dipalmitoyl-*sn*-glycero-3-phosphocholine (DPPC) liposomes (41 °C). However, when the temperature was increased to > 42 °C, the liposomes changed to be in a liquid crystalline state and the fluorescence intensity dramatically increased. This fluorescence switching at the phase transition temperature of the liposomes was in good agreement with previous reports of liposomes containing SQR22, which correspond to the PEG-TLip in this study^18^. The results showed that the attachment of biotin on the surface of the liposomes had little effect on the temperature-responsive fluorescence properties, indicating that the PEG-TLip can be used as a negative control for the biotin-TLip. The phase transition enhances the membrane bilayer permeability, fluidity, and mobility^16,17^ and the line tension, which describes the interfacial energy at the edge of the membrane domain or at the lipid phase separation^20^, between the lipid tails decreases from a few pN to 0 when the liposomes are transformed from the gel state to the liquid crystalline state^22–24^. When liposomes that contain SQR22 in the lipid bilayer membrane were exposed to temperatures lower than the critical temperature, the line tension of the pure DPPC regions was reported to be approximately 10–13 pN^25,26^. This line tension contributes to exclude the molecules loaded in the lipid bilayer, such as SQR22, from the DPPC region, which in turn increases the line tension^27^, resulting in the packing of the SQR22 molecules together and aggregation-caused quenching. When the liposomes are heated close to the phase transition temperature, the line tension drastically decreases and then vanishes according to the following law: λ(T) is proportional to (1 − T/T_c_), where T_c_ is the phase transition temperature of the liposomes and T ≤ T_c_^22^. This change in line tension suggests that the phase transition from gel to liquid crystalline states allows SQR22 to diffuse into the membrane bilayer and emit strong fluorescence. The temperature sensitivity of the TLip during the phase transition (41 to 45 °C) was 18.6% per °C, while fluorescein-27 and rhodamine B mixture only exhibits a temperature sensitivity of 4% per °C under 532 nm excitation wavelength^28^. These results indicated that biotin-TLip exhibited a remarkable increase in the fluorescence intensity at the phase transition temperature. Such fluorescence properties of biotin-TLip are a considerable advantage in designing a fluorescent probe for highly sensitive detection.

### Proof-of-concept study

**Figure 3:**
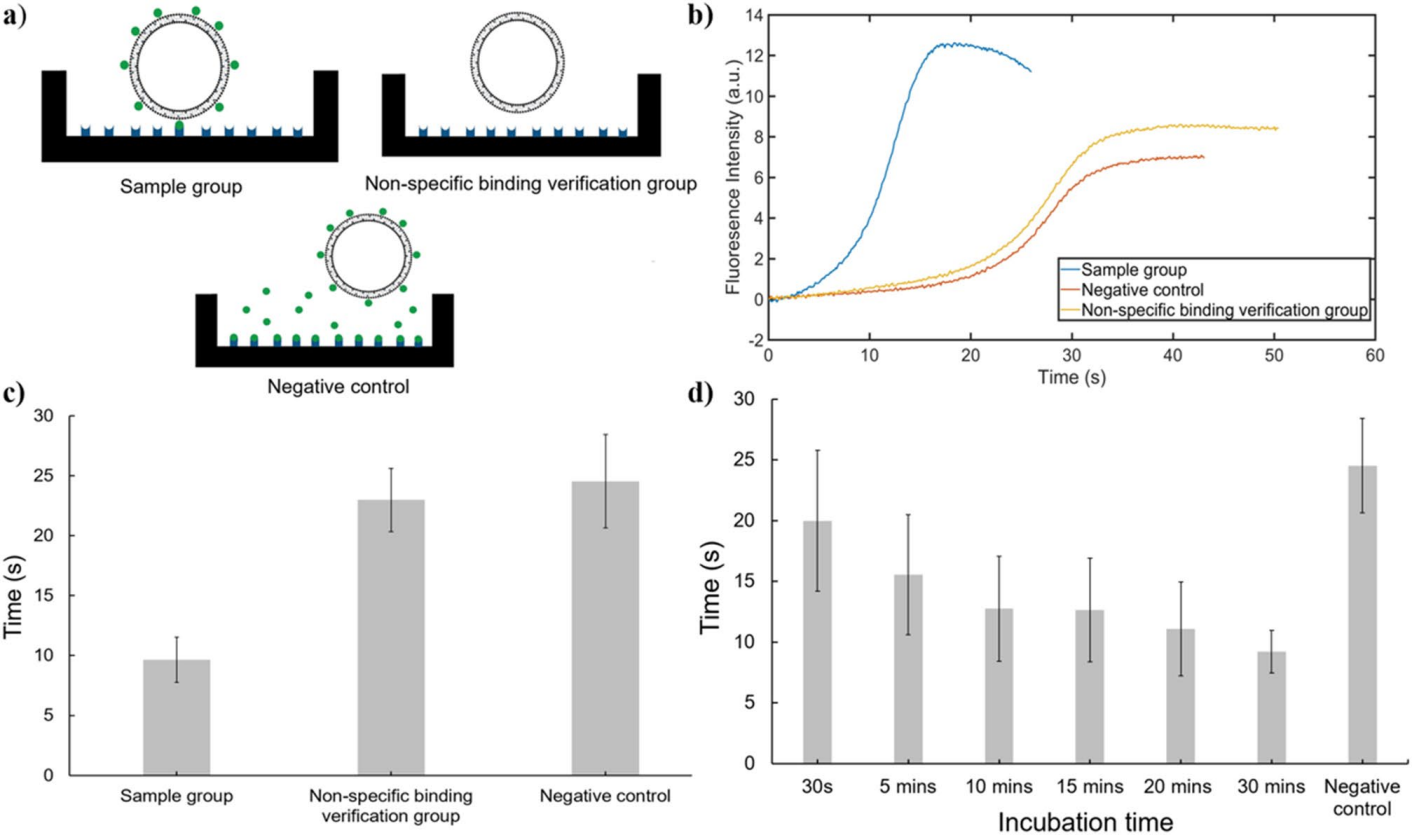
A proof-of-concept study using biotinylated TLip and a streptavidin-immobilized microwell plate. **(a)** Illustration of the bottom of the well for the different groups. The sample group contained biotin-TLip with a streptavidin-coated well. In the non-specific binding verification group, PEG-TLip was used instead of biotin-TLip and in the negative control, streptavidin was fully blocked by free biotin. **(b)** Example of results in biotin–streptavidin approach. **(c)** Time difference in three different groups, sample group n = 8, negative control n = 9, non-specific binding verification group n = 6. **(d)** Time difference in terms of incubation time, n = 9 for negative control, n = 4 for test groups.

The sample group, which involved biotin-TLip and non-treated coated streptavidin (Fig. 3a), was moved to a hot plate pre-heated to 80 °C. The fluorescence intensity change over time was then monitored by a micro-optic probe to determine the pattern of the rate of change, from an increase in increasing rate to a decrease in increasing rate, of the S curve as shown in Fig. 2, which was used as a key parameter to distinguish between negative and positive results. The non-specific binding verification group used PEG-TLip instead of biotin-TLip, and in a negative control, coated streptavidin was pre-blocked by free biotin solution (Fig. 3a), and biotin-TLip was diluted by a high concentration of biotin solution (100 μg/mL). The average times to reach the inflection point of the S-curve of the increase in the fluorescence intensity in the negative control groups (24.5 s) and non-specific binding verification groups (23.0 s) were similar (Fig. 3b,c). This result suggested that there was no non-specific binding between PEG-TLip and streptavidin and the plate; therefore, only biotinylated-PEG had the ability to allow binding between the liposomes and streptavidin proteins. In contrast, the sample groups showed an inflection point at 9.8 s. It was clear that there was a large time difference between the free and bound liposomes, and this time gap in the inflection points was calculated to be approximately 14 s. Such a remarkable time difference demonstrated that the presence or absence of target molecules, streptavidin in this case, greatly influenced the degree of the rate of increase in the fluorescence intensity of the liposomes. It took approximately 30 s for a 40-μL sample to reach 42 °C in the streptavidin-immobilized plate. This long time required to heat the free liposomes also showed the high heating efficiency of the bound liposomes to reach the phase transition temperature.

To evaluate the minimum incubation time for effective liposome binding, a number of samples were incubated for different times, 30 s, and 5, 10, 15, 20, and 30 min, a total of six groups. It required nearly 30 s to set up the whole system for fluorescence intensity change monitoring after adding the liposome samples. Each streptavidin-immobilized well was treated independently with 40 μL of liposome dispersion samples that had a concentration of 5 μg/mL SQR22 (the lipid concentrations of the biotin-TLip and PEG-TLip were 0.29 and 0.26 mg/mL, respectively). Figure 3d shows that as the incubation time increased, less time was needed for the liposomes to reach the inflection point of the fluorescence intensity change. The results indicated that because of the high binding rate constant (3.0 × 10^6^ − 4.5 × 10^7^ M^−1^ s^−1^)^29^ between biotin and streptavidin, a time of 10 min was sufficient for an effective number of biotin-TLip to bind to the coated streptavidin and emit fluorescence faster than the negative control under heating. However, as a large number of streptavidin molecules were bound by liposomes, the possibility of other liposomes binding to free streptavidin was greatly reduced. More importantly, the large size of the binding liposomes created spatial hindrance for other free liposomes to bind to streptavidin. Therefore, the results indicated that an incubation time of 10 min was sufficient for the ELISA-mimicking system to bind to the receptors and detect potential target biomolecules.

### LOD investigation by evaluating different time points

**Figure 4:**
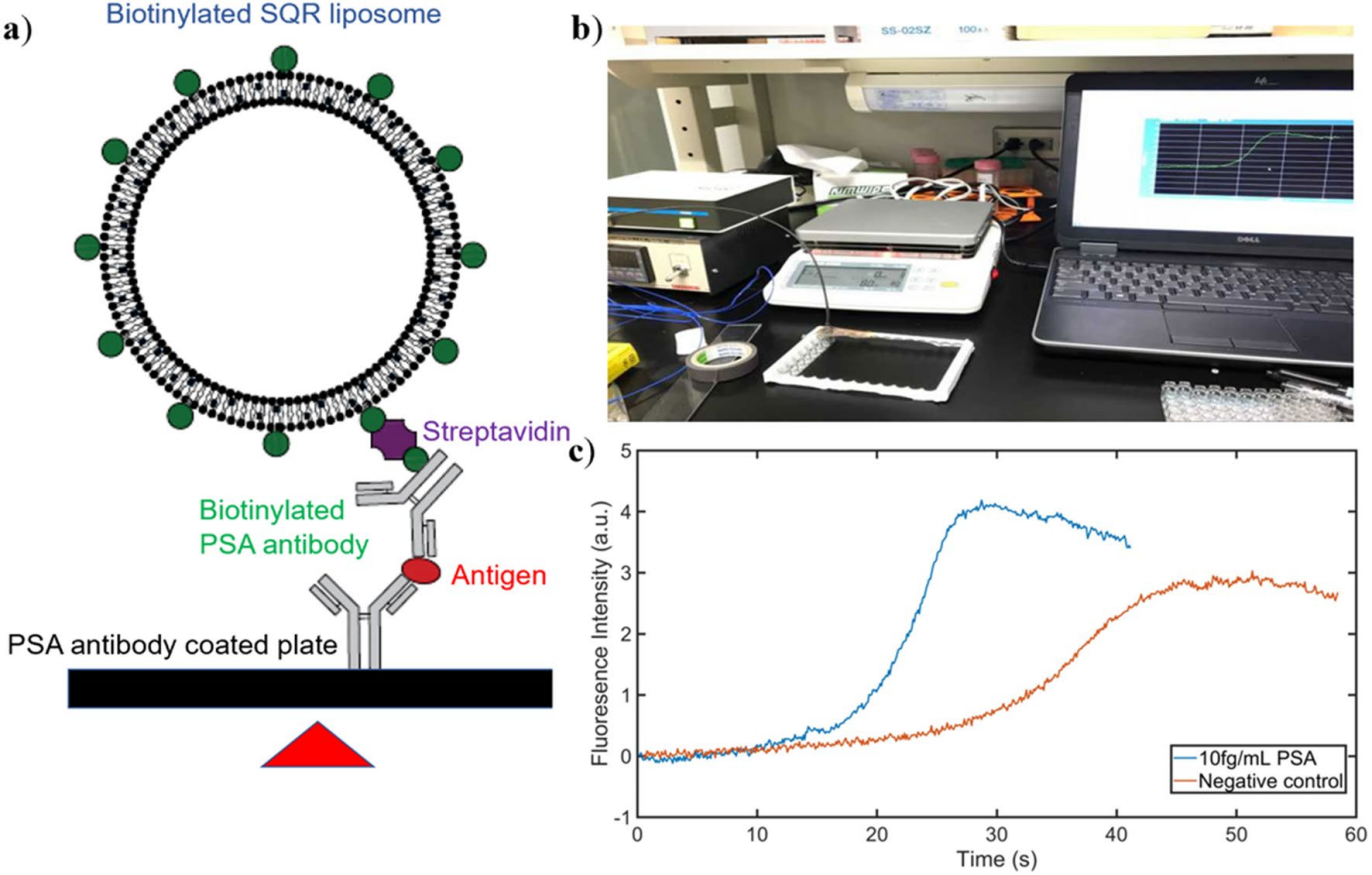
TLip-LISA demonstration using PSA. **(a)** Illustration of the sandwich-type complex to detect the antigen (PSA) with TLip. **(b)** Experimental set-up for monitoring the fluorescence intensity change after completion of the incubation steps. **(c)** Results for 10 fg/mL (350 fM) PSA and the negative control. Both samples showed an S-pattern increase, but the negative control took a longer time to reach the liquid crystalline state and emit fluorescence.

To examine the LOD of the TLip-LISA for PSA, PSA solutions with different concentrations were prepared from 10 pg/mL (350 fM) to 10 ag/mL (350 zM) with tenfold intervals. To form the complex shown in Fig. 4a, the PSA samples were first added to anti-PSA antibody-immobilized wells followed by addition of the biotinylated anti-PSA antibody for detection, and streptavidin and biotin-TLip. Each step had a specific incubation time and was followed by a washing step as described in the Methods section. The negative control group used DPBS, instead of PSA, and therefore no complex would form between the biotin-TLip and the anti-PSA antibody immobilized wells. All the liposome samples were diluted to a final SQR22 concentration of 5 μg/mL. After all the incubation procedures were completed, a micro-optic probe connected to the fluorescence detector, was attached to the target well and fixed with tapes and a hollow rubber cap as shown in Fig. 4b. Then, the antibody-immobilized strip was moved to a hot plate and the fluorescence intensity was monitored using the software associated with the fluorescence detector.

As shown in Fig. 4c, the presence of PSA (10 fg/mL) allowed the biotinylated SQR liposomes to bind to streptavidin and form complexes bound to the bottom of the well. This linkage permitted faster heat transfer from the hot plate to the bound liposomes, therefore, the liquid crystalline state was reached more rapidly, and fluorescence was emitted that could be detected with the micro-optic probe. In the negative control group, PEG-TLip cannot bind to either streptavidin or the substrate at the bottom of the well because of the absence of biotin and the presence of PEG on the liposomes. The temperature of the free liposomes was raised by the surrounding environment, which was water. As the heat capacity of water is high, the free liposomes required a longer time to reach the liquid crystalline state, which caused a time delay in the increase in fluorescence intensity compared with the 10 fg/mL (350 aM) PSA group.

### Investigation of the LOD of the TLip-LISA using PSA as the target molecule

**Figure 5:**
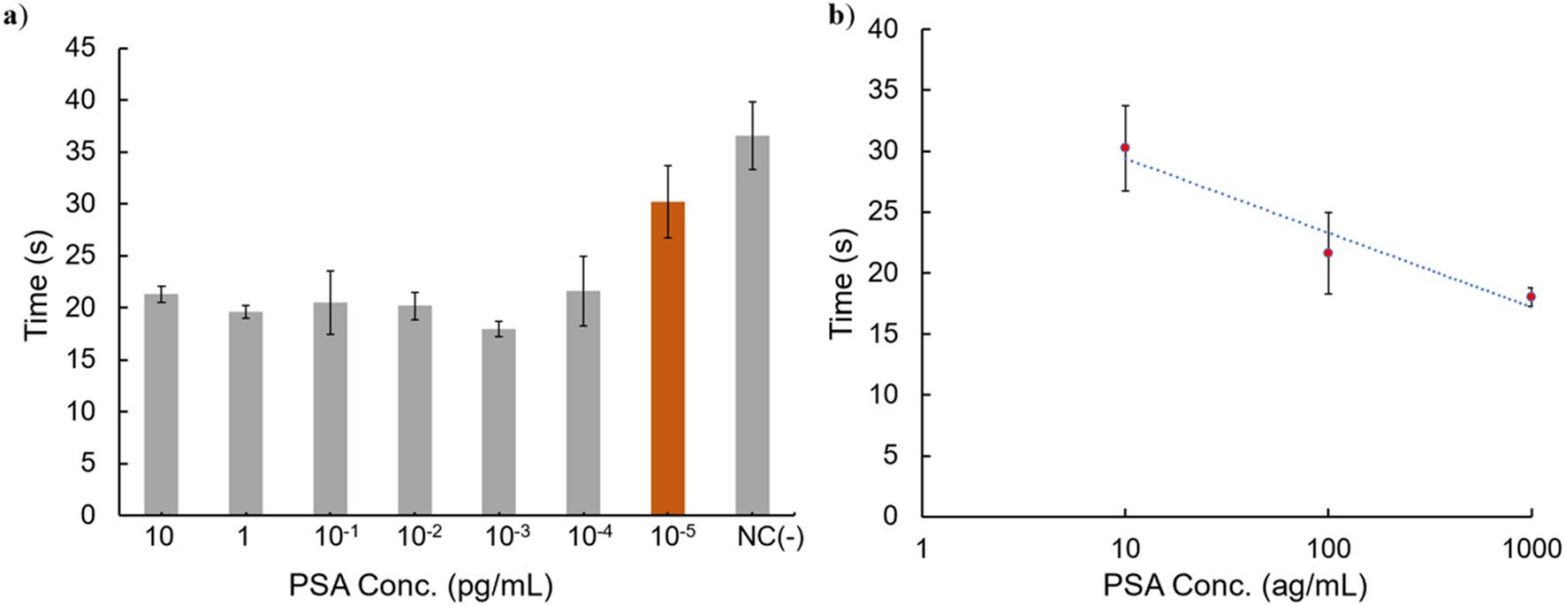
Detection of PSA from the difference in the inflection time point of fluorescence intensity change. **(a)** Time to reach the inflection point of the change in the fluorescence intensity of biotin-TLip for various PSA concentrations, n = 3. NC is negative control with no PSA added. **(b)** Linear regression results between PSA concentrations (1 fg/mL to 10 ag/mL) and inflection time.

As shown in Fig. 5a, it took nearly 36 s for the liposomes in the negative control group to reach the inflection point of the change in the fluorescence intensity, while the time for the groups with PSA concentrations ≥ 100 ag/mL (3.5 aM) was approximately 15 s faster. The time to reach the inflection point where increase rate reaches maximum is estimated to be corresponding to the time to reach the heating of TLip around 43 °C where the change of fluorescence intensity per temperature is maximum in Fig. 2. The considerable inflection time difference between positive and negative control groups implied that the absence or presence of target molecules could greatly influence the time at which the liposomes started to convert to the liquid crystalline state. When the PSA concentration was 10 ag/mL, the average time was 30.2 s, which was only slightly different from the negative control group. The LOD was determined by extrapolating the concentration from the inflection time equal to negative control inflection time minus three times standard deviation of negative control. The LOD of our system in PSA detection was calculated to be 27.6 ag/mL, 0.97 aM.

## Discussion

Our research is the first study to use temperature-responsive fluorescent liposomes as signal generators and amplifiers to detect target biomarkers. Previous studies which utilize liposomes as platform do not exhibit such unique optical property that signal intensity of liposomes, not targets, will drastically change under different circumstances, which is temperature in our case^30–32^. We used a special parameter, inflection time point, to identify the presence or absence of target molecules instead of fluorescence intensity difference as bound and unbound liposomes emit fluorescence at different time points under heating. The results presented here indicated that the time difference was a crucial factor to detect the presence of target molecules using the TLip-LISA. By employing SQR liposomes as a detection tool, the LOD of the TLip-LISA for PSA detection was 27.6 ag/mL (0.97 aM), which was nearly 300,000-fold more sensitive than conventional ELISA tests regarding the mass concentration, as conventional ELISA methods showed no clear differences in signals below 100 pg/mL of PSA (see Supplementary Table S2, Fig. S2 online). Our data suggest that SQR liposomes were able to greatly improve the sensitivity of the current detection technology with high reliability.

The response time for conventional ELISA tests is nearly 30 min, because the fluorescence intensity depends on the enzymatic digestion period^33^. Digital counting ELISA methods also need a long incubation, up to 5–6 h, to enhance the signal-to-noise ratio^14,15^ to allow effective signal reading. Our system, however, does not need incubation for a long time to enhance the signal intensity for effective reading. This shorter time is because one liposome can carry an average of approximately 2000 SQR22 molecules (see Supplementary Table S1 online), and each SQR22 molecule can emit photons as signals when the liposome reaches the liquid crystalline state. Hence, the liposome itself can act as a signal amplifier to enhance the fluorescent signals. Therefore, our system does not require a long incubation period to obtain effective signals for analysis and thus, the response time is less than 1 min, which is the time required for the liposomes to undergo phase transition. Additionally, from the results of the proof-of-concept study, our TLip-LISA also can reduce the incubation time for complex formation. In the proof-of-concept study, the required incubation time could be shortened to 10 min. This short incubation time is probably because of the high binding rate between biotin and streptavidin that leads to a substantial number of bound liposomes in a short time to promote a faster fluorescence response under heating. A previous report has shown that biotinylated DNA and Alexa Fluoro 488-conjugated streptavidin formed complexes in seconds in micro-droplets and emitted effective signals when induced by a green laser^29^.

The principle underlying the detection technology is also different from current ELISA methods from another perspective. We used a detection tool that only emits fluorescence above a certain temperature (lipid-dependent), whereas current ELISA methods use enzymes to digest substrates to obtain signals based on the amount of the product. This dissimilarity in fluorescence generation principle between our system and ELISA methods causes differences in the analysis regarding the judgement of positive and negative results. For example, conventional ELISA tests are analog measurements, which means the fluorescence intensity increases as the concentration of the target molecule increases^15^. The ELISA methods, therefore, are able to present a linear relationship between the target molecules and the absorbance over a specific range in the post-analysis. However, the background noise is high, which presents difficulties in identifying the presence of target molecules. Our system, however, uses a completely different strategy to distinguish between positive and negative samples. The presence of target molecules allows surface-modified TLip to form a complex between an antibody-immobilized well, a biomarker, and a detection antibody. In the absence of target molecules, the SQR liposomes cannot form a complex and link to the antibody-immobilized plate through the secondary antibody and target molecule. The bound liposomes can directly acquire heat from the bottom of the plate and rapidly reach a liquid crystalline state when the plate is heated, while the temperature of the free liposomes depends on water, which has a high heat capacity. The observed time differences in the effective fluorescence emission enabled a simple and universal way to determine positive and negative samples, whereas ELISA methods need statistical analysis to avoid false-positive or false-negative results in different applications. The underlying mechanism of TLip-LISA, thus, can provide temporal and financial savings in establishing tests for new biomarkers.

Both the PSA group and the negative control showed similar S-curve patterns in the fluorescence intensity over time (Fig. 4c). It was expected that the fluorescence intensity changes of the positive samples would show a two-step increase when free liposomes were not washed, considering the domination of free liposomes (the number of free liposomes was approximately 3.66 × 10^11^, while of the number of bound liposomes was approximately 2 × 10^7^ at a PSA concentration of 10 pg/mL). It is possible for liposomal dispersions under heating to show a two-step increase if the numbers of free liposomes and bound liposomes are similar, and to show a one-step increase, from the signals of the bound liposomes, if the majority of the liposomes are bound to the bottom of the well. In case of a two-step increase, the first increase is because of the heating of the bound liposomes, followed by a gentle increase originating from the free liposomes transforming to the liquid crystalline state. However, the result curve of 10 fg/mL PSA in Fig. 4 c showed a one-step pattern, which appeared earlier than the control over the whole observation. This result possibly can be explained by the rapid exchange of bound liposomes with free liposomes because the biotin–streptavidin interaction can be reversibly broken in water, particularly at high temperatures^34^. A previous study has shown that a single water molecule can enter the binding pocket, where it acts as bridge between the Asp-128 of streptavidin and biotin, which results in disassociation of biotin from the biotin–streptavidin binding pocket and this bond-breaking event can occur in water at an elevated temperature^34,35^. Therefore, the bound liposomes are first heated above the liquid crystalline state to emit fluorescence and then are released from the complex because of the increased dissociation rate at high temperatures. The surrounding free liposomes then can bind to streptavidin to form new complexes and then quickly reach the liquid crystalline state to emit fluorescence and be released by the break-up of the complexes. Such an exchange cycle would be repeated highly efficiently, so that the pattern of the fluorescence intensity increase is manifested continuously not discretely. This cycle can explain why the system showed a high fluorescent intensity change even with small amounts of antigen. Antibodies are known to denature at high temperatures, for example, immunoglobin G has been shown to be completely denatured within 20 min at 70 °C^36^. The unfolding of the denatured antibody results in the antibody losing the high affinity for antigens, leading to the loss of bound liposomes and a longer time to reach maximum fluorescence. However, the antibody-immobilized wells and detection antibodies were heated from room temperature with a high heating rate and the heating time was less than 1 min, which might reduce the effect of denaturation considerably.

Based on the signal acquisition difference, TLip-LISA does not require a large and expensive micro-plate reader to measure the signal intensity as a sandwich ELISA method do. Instead, TLip-LISA used a much smaller, convenient and cheaper fluorescence detector to read the signal for further analysis. The low price and portability of desired equipment of TLip-LISA permit underdeveloped area to use this assay, or carried by physicians, for fast and accurate early-diagnosis. In this study, the TLip-LISA method successfully detected PSA at 100 ag/mL, with a LOD of 27.6 ag/mL, the sensitivity was remarkably higher than the conventional ELISA methods, which only can detect PSA at 8 pg/mL according to manufacturer’s protocol. TLip-LISA utilizes capture and detection antibodies to target biomarkers highly specifically and selectively as sandwich ELISA method. However, we did not use clinical samples to validate the specificity of TLip-LISA. We will test in the future experiments to prove the specificity of TLip-LISA for potential clinical use. The incubation procedures of our method were similar to the sandwich ELISA method, except we used SQR22 liposomes instead of digestion enzyme to generate signals for read-out. The incubation time in this study followed the conventional ELISA method, but could potentially be reduced according to the results of proof-of-concept study. These similarities between TLip-LISA and sandwich ELISA make TLip-LISA user-friendly and allows researchers and practitioners in clinical fields to quickly accept and be accustomed to use it in any biomarker detection. TLip-LISA also did not need new designed plates or devices, but regular 96 well plate with immobilized capture antibodies. On the contrary, digital ELISA methods require a plate contains hundreds of thousands of microwells and corresponding beads with immobilized antibodies to enable high sensitivity^13,14^. Another example is Macchia et al. designed a millimetre-meter sized transistor to detect human IgG at single molecule level^37^. Those new devices increase financial and learning cost for other people to use. On the other hand, TLip-LISA showed much higher sensitivity compared to other methods using liposomes as platform to carry fluorescent dyes, which only exhibited sensitivity no higher than 1 pM^30,38,39^. In this study, PSA detection results were obtained from three independent measurements, where stability and reproducibility need to be further investigated since conventional and digital ELISA methods use much more numbers of wells to reduce errors and improve accuracy^13,14,33^.

Our system still has some limitations to be improved. First, although our system manifested a high sensitivity for the target molecules, the fluorescence intensity was unable to be used for quantification analysis. Because the concentration of SQR22 was the same for each tested liposomal sample, the highest fluorescence intensity of each sample was also similar. In addition, the position of micro-optic probe may vary as it is required to be pressed to be better fixed on a target well, with the help of tapes, which may lead to fluorescence intensity differences. These factors are obstacles for the TLip-LISA to be used for quantification analysis, in terms of fluorescence intensity. In contrast, the concentration of target molecules can be obtained by analyzing the results of conventional ELISA tests and digital counting ELISA methods. However, quantification analysis might be possible between 1 fg/mL to 10 ag/mL, as over this concentration range there was potentially a linear relationship between the time and the PSA concentration, as shown in Fig. 5b. Therefore, further investigation of PSA concentrations between 1 fg/mL and 10 ag/mL is needed for possible quantification analysis. Secondly, though the liposomes exhibited a narrow size distribution, the size difference between the smallest and largest particles can be up to 60 nm in diameter, and a much larger difference in terms of volume. Larger liposomes can carry more SQR22 than smaller ones, and thus provide higher fluorescence intensity. This effect is another impediment for quantification analysis. Lastly, conventional and digital ELISA methods can detect 96 samples at one time, while our system only monitors one well per measurement, along with requiring micro-optic device plug-in and -out. This inefficient procedure is not user-friendly and greatly hinders any industrial use. We are now developing the system to detect the fluorescent intensity changes of 96 samples using a highly sensitive camera and a thermoplate. In conclusion, TLip can be used in a biomolecular detection system and our TLip-LISA showed high sensitivity and good reliability with a fast response for PSA detection. Our system successfully detected PSA at 100 ag/mL in a 100-μL sample, which was approximately 10^6^-fold more sensitive than conventional ELISA tests in terms of mass concentration, and showed a remarkable difference with the negative control. The LOD of our system was determined to be 27.6 ag/mL, 0.97 aM. Our results indicated that TLip-LISA has the potential to improve on current biomolecular detection technology, and will be of benefit for diagnostic and biological inspection testing, such as an antigen detection system for new coronavirus (COVID-19).

## Methods

### Materials & reagents

The following reagents and materials were purchased from Sigma-Aldrich, USA: streptavidin and a PSA ELISA kit, including biotinylated anti-PSA-antibody for detection, PSA molecules, and a kit of 96 wells (12 strips × 8 wells) to which anti-PSA-antibody for capture was immobilized. 1,2-Distearoyl-*sn*-glycero-3-phosphoethanolamine-*N*-[biotinyl(polyethylene-glycol)-2000] (biotinylated-PEG_2000_-DSPE) was purchased from Avanti Polar Lipids, Inc. (Alabaster, AL, USA). Other lipids used to prepare liposomes were DPPC, DHSG and PEG_5000_-DSPE. DPPC and DHSG were purchased from Nippon Fine Chemical (Tokyo, Japan), while PEG_5000_-DSPE was purchased from NOF Corporation (Tokyo, Japan). The fluorescent dye SQR22 was synthesized according to a previous report^18^. *Tert*-butyl alcohol (*t*-BuOH) was used to lyophilize the lipids and was purchased from Kanto Chemical (Tokyo, Japan). Dulbecco's phosphate-buffered saline (DPBS) was used as a solvent and was purchased from Thermo Fisher Scientific (Waltham, MA, USA). The phospholipid assay kit was obtained from FUJIFILM Wako Pure Chemical Corporation (Osaka, Japan). Octylglucoside (OG) was purchased from Carbosynth, (Berkshire, UK).

### Liposome preparation

Biotin-TLip were composed of DPPC, DHSG, biotinylated-PEG_2000_-DSPE (PEG_5000_-DSPE was used to prepare PEG-TLip), and SQR with a molar ratio of 86.6:9.6:0.5:3.3. The mixed lipids were initially dissolved in *t*-BuOH and transferred to a 30-mL flask. The mixed lipid solution was freeze-dried by using a freeze-dryer (Yamato, Japan) overnight. The obtained mixed lipid powder was hydrated with DPBS at 3 mg/mL. Then, the lipid dispersion was mixed using a vortex mixer (Scientific Industries, USA) for 15 min. After vortex mixing, the dispersion was passed through hydrophilic membrane filters (Pore sizes: 0.45 μm × 1, 0.2 μm × 1, 0.1 μm × 1, and 0.05 μm × 3; Sigma-Aldrich, USA) using an extruder (Norther Lipids Inc., Canada) with applying nitrogen gas pressure to obtain a monodispersed size distribution.

### Characterization of liposomes

To measure the mean diameter and size distribution of the liposomes, 10-μL samples from the extruded liposome dispersions were diluted in 990 μL of DPBS solution, and this sample (a total of 1 mL) was loaded in a plastic cuvette. The size and poly dispersity index of the liposomes were measured by a Zetasizer (Malvern Zetasizer Nano S90, Malvern Instruments Ltd., UK) using the dynamic light scattering (DLS) technique. Then, the same sample was transferred to a capillary cell mounted on the apparatus for zeta potential measurement. The zeta potential of the liposomes was measured using the Zetasizer according to the manufacturer’s instructions. All the measurements were conducted three times. The concentration of the lipids was determined with a phospholipid C kit (FUJIFILM Wako Pure Chemical Corp. Japan) according to the manufacturer’s instructions. The standard curve of SQR22 was constructed from the absorbance of SQR22 at 631 nm for different concentrations of SQR22 in ethanol. The concentration of SQR22 in the liposomes was then calculated from the standard curve.

### Verification of temperature-responsive fluorescence

After determination of the SQR22 concentration in the liposome dispersions, a liposomal sample with 1 μM SQR22 was prepared by DPBS dilution. The sample was transferred to a transparent quartz cuvette and the fluorescence intensity of the liposomes was measured (λ_ex_ = 570 nm, λ_em_ = 660 nm) at different temperatures, ranging from 25 to 60 °C with 5 °C intervals, by a fluorescence spectrophotometer (RF-5300PC, Shimadzu Corporation, Japan). The temperature of the liposome dispersion was controlled by water flow, and the inlet water-flow was heated by a water bath at a constant heating rate (3.4 ± 0.55 °C/min) to 60 °C. The liposomal dispersion temperature was monitored by a digital thermometer (CT-800WP, Custom, Japan) immediately before intensity measurement. The fluorescence intensity around the phase transition temperature of the DPPC liposomes (40–43 °C) was measured at 1 °C intervals.

### Proof-of-concept system experimental set-up

A streptavidin-immobilized 96-well plate (Thermo Scientific, USA) was used for the proof-of-concept study. Biotin-TLip were used in the sample groups to observe a faster fluorescence intensity change. In the negative control, streptavidin was previously blocked by a free biotin solution (50 μg/mL, 150 μL). The negative control was used to measure the fluorescent intensity change of free liposomes. In the non-specific binding verification group, liposomes consisting of PEG_5000_-DSPE (PEG-TLip), instead of biotinylated-PEG_2000_-DSPE, were added to a streptavidin-immobilized well to examine whether there was any non-specific binding between the liposomes and streptavidin. The SQR concentration in all the groups was set at 5 μg/mL by dilution. A hot plate (RSH-1DR, AS-One, Japan) was used to provide heat. The hot plate was pre-heated to 80 °C to provide steady heating of the streptavidin-immobilized 96-well plates. Before placing the immobilized-plate on the heating device, 40 μL of liposomal samples were added to the wells and incubated at room temperature (27 °C) for a set incubation time to form the biotin–streptavidin complex prior to heating. Then, the immobilized-plates were placed on the hot plate and a fluorescence detector (FLE1100, Nippon Sheet Glass Co. Ltd., Japan) with a micro-optic probe with a working distance of 10 mm and spot size of 1.0 mm (Probe40100) was used to monitor the fluorescence intensity change versus time. To fix the micro-optic probe, a rubber cap was fabricated to be hollow to allow the probe to go through and a rubber ring was used to help maintain the probe at a specific position (see Supplementary Fig. S1 online). Then, single-sided tapes were used for stabilizing the micro-optic probe during monitoring. The results were evaluated by the time to reach the highest fluorescence intensity.

To assess the minimum incubation time required to form the biotin–streptavidin complex that enabled effective and fast fluorescence emission from biotin-TLip, multiple 40-μL liposomal samples were incubated in the streptavidin-immobilized wells for 30 s, and 5, 10, 15, 20, and 30 min. The results were compared with the negative control to determine the minimum incubation time required.

### LOD investigation of the TLip-LISA

To investigate the LOD in our system, PSA samples with different concentrations were prepared, from 10 pg/mL to 1 ag/mL with tenfold intervals. The biotinylated antiPSA monoclonal antibody for detection was diluted by 80-fold in DPBS according to the manufacturer’s protocol, and streptavidin was dissolved in DPBS and diluted to 2.5 μg/mL for use. To form the complex between the liposomes and anti-PSA monoclonal antibody-immobilized wells (used as purchased 12 strips × 8 wells) in the presence of PSA, 100 μL of PSA solution was first added to the well and incubated for 2.5 h with gentle shaking (Shaker SRR-2, AS-ONE, Japan). Then, the unbound PSA was washed out by 3 × 100 μL of DPBS. The biotinylated anti-PSA-antibody (100 μL) for detection was then added to the same well and incubated for 1 h with shaking and a washing step (100 μL DPBS, 2 ×) after incubation. After that, the 100 μL of streptavidin solution with a concentration of 2.5 μg/mL was added to the target wells and incubated for 45 min with gentle shaking. Then, the free reagents were washed out by 2 × 100 μL of DPBS. Finally, 60 μL of TLip dispersion with a SQR22 concentration of 5 μg/mL was added to the well followed by 30 min of incubation.

Similar to the proof-of-concept experiments, we used the same hot plate, which was pre-heated to 80 °C, which promoted fast heating of the bound liposomes, and the same fluorescence detector to monitor the fluorescence intensity changes of the biotin-TLip. To better compare the time differences caused by the PSA, the negative control groups no longer used PEG-TLip. Instead, biotin-TLip were used in the negative control group, but no PSA molecules were added in that group. We used a micro-optic probe with a working distance of 2 mm and spot size of 0.2 mm (Probe4020) for higher precision and sensitivity. The results of the fluorescence intensity changes were evaluated in terms of the inflection point of the fluorescence intensity change.

### Calculation of LOD

The calculation method of LOD in this study was based on previous highly sensitive detection methods^13,14,37^. A linear regression line of PSA concentration and inflection time was obtained between 1 fg/mL and 10 ag/mL. The LOD was computed as the PSA concentration corresponded to the inflection time equal to the average inflection time of negative control minus three times of the negative control standard deviation. The average inflection time of negative control was 36.6 s with standard deviation of ± 3.3 s. Thus, the computed LOD was determined to be 27.6 ag/mL corresponding to an inflection time point of 26.7 s.

## Supplementary information

Supplementary Information.
