# Supplementary material for: A rapid and highly sensitive biomarker detection platform based on a temperature-responsive liposome-linked immunosorbent assay
Source: Sci Rep. 2020 Oct 22;10:18086. doi: 10.1038/s41598-020-75011-x (PMC7582967; doi:10.1038/s41598-020-75011-x)
Supplement: Supplementary file 1 — Supplementary Information. [file 41598_2020_75011_MOESM1_ESM.pdf]

Supporting information

A rapid and highly sensitive biomarker detection platform based on a temperature-responsive liposome-linked immunosorbent assay

Runkai Hu, Keitaro Sou*, Shinji Takeoka*

*Corresponding authors. Email: soukei@aoni.waseda.jp, takeoka@waseda.jp

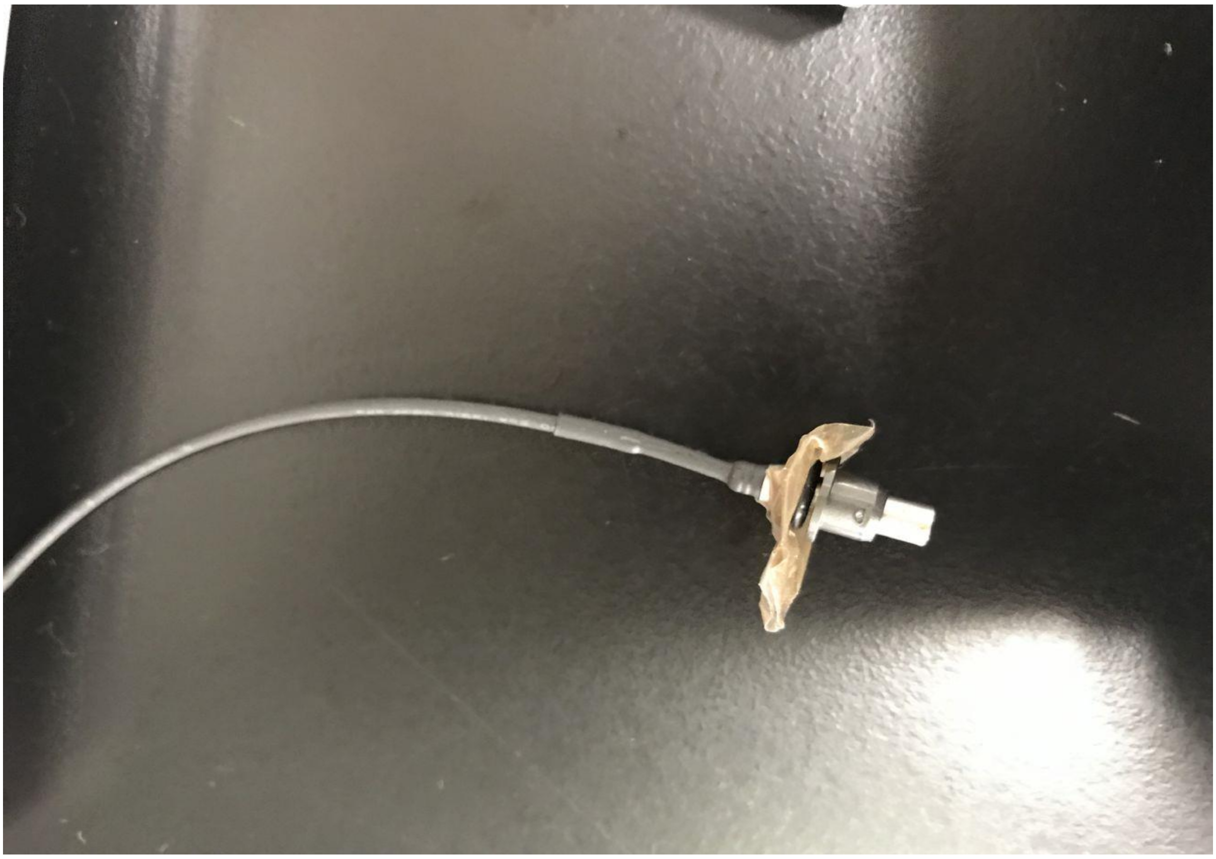

Figure S1. Tools for fixation of micro-optic

$$\boldsymbol{N_{tot} = 4\pi \frac{(\frac{d}{2})^2+(\frac{d}{2}-h)^2}{A}} \tag{S1}$$

Equation S1[1]. This equation is used to approximate average number of lipids to form a liposome, where d is average mean diameter of liposome, h is thickness of bilayer. A is area of phospholipid head (phosphatidylcholine) and N_{tot} is number of lipids needed to form one liposome

$$\boldsymbol{N_{lipo} = \frac{M_{lipid} * N_A}{N_{tot}}} \tag{S2}$$

Equation S2[1]. This equation is used to calculate total number of liposomes given lipid concentration and volume of liposomal dispersion, where M_{lipid} is molar concentration of total lipids, N_A is Avogadro constant. N_{lipo} is total number of liposomes.

The total number of liposomes were calculated according to **Equation S1 & S2.** The number of PSA was calculated based on its molecular weight [2] and concentration in a 100 µL sample. The maximum number of bound liposomes were calculated based on number of PSA. The number of SQR22 molecules were obtained based on number of total lipids to form one biotin-TLip and its molar ratio after extrusion (2.8%). The number of SQR22 varied because liposome size ranged from 64.4 to 124.6 nm (**Table 1**). The average number of SQR22 molecules in one liposome obtained from mean size of biotin-TLip, which was 1991.

Table S1. Number of reagents based on size of biotin-TLip. Number of PSA was concentration dependent.

Reagent Name	Approximate Number	Unit
Free liposomes	3.66×10^{11}	60 μL
PSA	$200\text{-}2\times10^{7}$	100 μL (from 100 ag/mL to 10 pg/mL
Max number of bound liposomes	$200\text{-}2\times10^{7}$	Corresponding to number of PSA
SQR molecules	880-3550	In one liposome

Table S2. ELISA absorbance data of PSA at different concentrations.

PSA conc. (pg/mL)	1000	250	100	10	5	1	0.1	Negative control
Abs	1.092	0.406	0.189	0.173	0.109	0.111	0.096	0.056
Abs	1.126	0.403	0.185	0.172	0.11	0.11	0.095	0.066

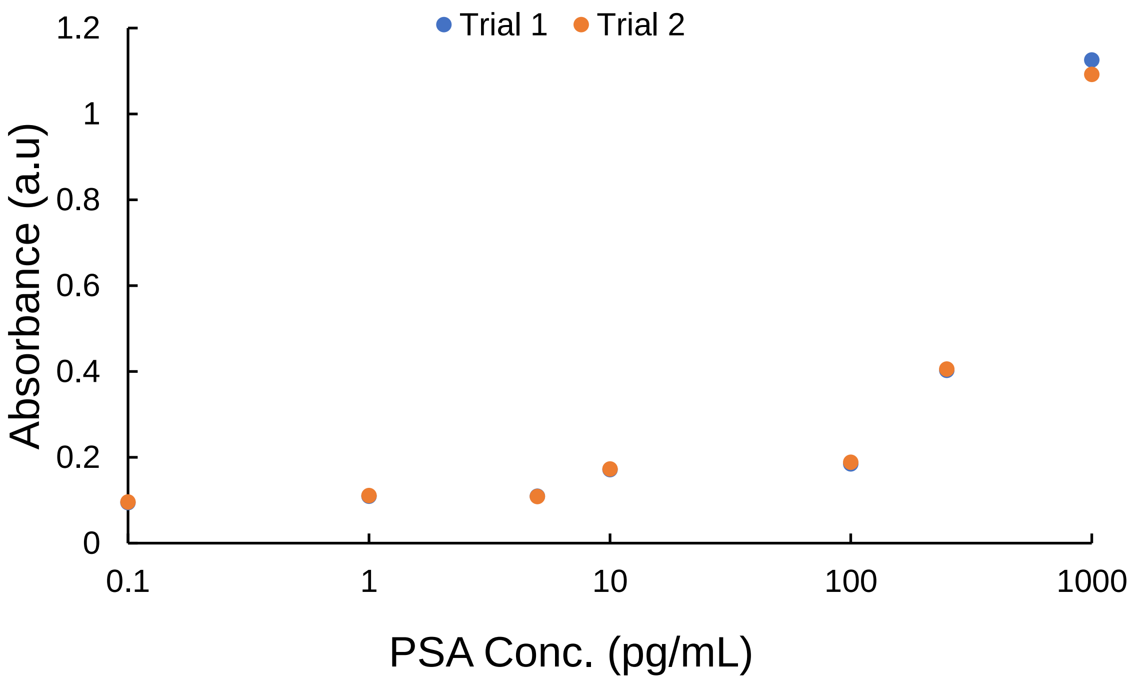

Figure S2. Results of PSA ELISA data (n=2). The absorbance showed linear relationship with PSA concentrations above 10 pg/mL, while intensity was almost no difference for PSA concentration below 10 pg/mL

References:

1. The Number of Lipid Molecules per Liposome. (n.d.). Retrieved July 29, 2020, from http://www.liposomes.org/2009/01/number-of-lipid-molecules-per-liposome.html.

2. Bélanger, A., et al. Molecular mass and carbohydrate structure of prostate specific antigen: Studies for establishment of an international PSA standard. *The Prostate*, **27(4)**, 187-197. doi:10.1002/pros.2990270403. (1995).
